# A Fast Online Replanning Algorithm Based on Intensity Field Projection for Adaptive Radiotherapy

**DOI:** 10.3389/fonc.2020.00287

**Published:** 2020-03-03

**Authors:** Xiaomeng Liu, Yueqiang Liang, Jian Zhu, Gang Yu, Yanyan Yu, Qiang Cao, X. Allen Li, Baosheng Li

**Affiliations:** ^1^School of Medicine and Life Sciences, University of Jinan-Shandong Academy of Medical Sciences, Jinan, China; ^2^Department of Radiation Oncology, Shandong Cancer Hospital and Institute, Shandong First Medical University and Shandong Academy of Medical Sciences, Jinan, China; ^3^Software Research and Development Department, STFK Medical Device Co, Ltd., Zhangjiagang, China; ^4^Shandong Key Laboratory of Medical Physics and Image Processing, School of Physics and Electronics, Shandong Normal University, Jinan, China; ^5^Department of Radiation Oncology Physics and Technology, Shandong Cancer Hospital and Institute, Shandong First Medical University and Shandong Academy of Medical Sciences, Jinan, China; ^6^Department of Neurology, Shandong Provincial Qianfoshan Hospital, Jinan, China; ^7^Laboratory of Image Science and Technology, Southeast University, Nanjing, Jiangsu, China; ^8^Department of Radiation Oncology, Medical College of Wisconsin, Milwaukee, WI, United States

**Keywords:** online replanning, adaptive radiotherapy, interfractional variations, image guided radiotherapy, deformable image registration

## Abstract

**Purpose:** The purpose of this work was to propose an online replanning algorithm, named intensity field projection (IFP), that directly adjusts intensity distributions for each beam based on the deformation of structures. IFP can be implemented within a reasonably acceptable time frame.

**Methods and Materials:** The online replanning method is based on the gradient-based free form deformation (GFFD) algorithm, which we have previously proposed. The method involves the following steps: The planning computed tomography (CT) and cone-beam CT image are registered to generate a three-dimensional (3-D) deformation field. According to the 3-D deformation field, the registered image and a new delineation are generated. The two-dimensional (2-D) deformation field of ray intensity in each beam direction is determined based on the 3-D deformation field in the region of interest. The 2-D ray intensity distribution in the corresponding beam direction is deformed to generate a new 2-D ray intensity distribution. According to the new 2-D ray intensity distribution, corresponding multi-leaf collimator (MLC), and jaw motion data are generated. The feasibility and advantages of our method have been demonstrated in 20 lung cancer intensity modulated radiation therapy (IMRT) cases.

**Results:** Substantial underdosing in the CTV is seen in the original and the repositioning plans. The average prescription dose coverage (V100%) and D95 for CTV were 100% and 60.3 Gy for the IFP plans compared to 82.6% (*P* < 0.01) and 44.0 Gy (*P* < 0.01) for original plans, 86.7% (*P* < 0.01), and 58.5 Gy (*P* < 0.01) for repositioning plans. On average, the mean total lung doses were 12.2 Gy for the IFP plan compared to the 12.4 Gy (*P* < 0.01) and 12.6 Gy (*P* < 0.01) for the original and the repositioning plans. The entire process of IFP can be completed within 3 min.

**Conclusions:** We proposed an online replanning strategy for automatically correcting interfractional anatomy variations. The preliminary results indicate that the IFP method substantially increased planning speed for online adaptive replanning.

## Introduction

Adaptive radiotherapy (ART) has the potential to correct for interfractional variations during radiotherapy (1, 2) by imaging the patients with technologies such as the CT on-rails or cone beam computed tomography (CBCT). However, online ART has not been widely used. Image guided radiotherapy (IGRT) is the current standard practice to account for interfractional variation, which is largely limited by rigid-body matching and is not able to fully correct for anatomic deformations (3–5). To fully correct for the interfractional anatomic variations, it is necessary to re-contour the target and organs at risk (OARs) based on the patient's daily image. To generate an adaptive plan, the replanning process including re-contouring, plan optimization and quality assurances needs to be completed in a short period of time. Otherwise, anatomical motion during the replanning time may offset the advantage of online ART.

In the last decade, researchers have made a great deal of effort to develop algorithms to speed up the replanning process. Ahunbay et al. proposed a two-step algorithm, SAM+SWO, which firstly adjusted the leaf position according to the changes in the target area, and then re-optimized the aperture weights to achieve the goal of quickly modifying the plan (6). Based on the SAM, they further introduced an online adaption method in flattening-filter-free beams (7). A method similar to SAM was virtual couch shift (VCS) technique. It actually rotates and converts the patient to find the best projection aperture that the MLC blade needs to adapt (8). Fredriksson used the dose distribution of the previous plan to constrain the new plan, by enforcing that the dose volume histograms (DVHs) of each voxel or region of interests (ROI) to be at least as good as the previous plan, and optimizing the OARs at the same time (9). Kontaxis et al. described a novel real-time adaptive treatment method where intrafraction, inter-beam re-planning and optimization takes place, taking into account the previously delivered dose within that fraction accumulated onto the underlying moving anatomy (10). Besides, magnetic resonance imaging (MRI) guided radiotherapy could also been used for daily online replanning. Yet, the anatomical structures re-contouring and replanning under MRI would expense of a longer treatment period (11). A more advanced method aims at generating a treatment plan based on the image of the day using the initial simulation treatment plan as a starting point (warm-start optimization) (12). This approach minimizes computational costs and is expected to mitigate some of the issues related to quality control and plan approval (13).

In this work, we proposed a fast online replanning algorithm named intensity field projection (IFP). The main idea is to make the ray in different directions following the changes in the aperture, realizing adaptive correction by projecting a 3-D vector field into a 2-D vector field for deformation of the intensity distribution. In this way, the adaptive plan would be superior to the original plan. The process utilizes a 3-D image registration algorithm that we previously proposed, named the gradient-based free form deformation (GFFD) algorithm (14). GFFD can automatically propagate the contours from the planning CT based on the CT of the day, thus, dramatically reducing human effort and replanning time.

## Methods and Materials

### The Overall Replanning Scheme

**Figure 1:**
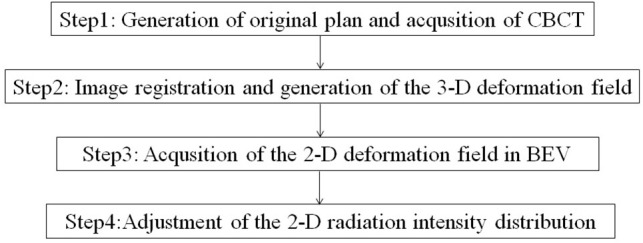
Flowchart of the IFP methodology.

Figure 1 displays the major steps of the IFP algorithm. Automatic contour and image registration were completed by software based on the GFFD algorithm. It has been reported that the GFFD algorithm can effectively complete the deformation registration of CBCT and CT images, while the target and organ contours were automatically propagated from the planning CT to CBCT (14). To further reduce the replanning time, we implemented the registration algorithm on a GPU through Open CL parallel programming.

### Generation of the Original Plan and Acquisition of CBCT

The original plan was generated using conventional planning techniques, such as IMRT, to achieve the desired dose-volume objections for the target and OARs based on the planning CT. Daily CBCT images were acquired immediately before delivery of the fractional dose.

### Image Registration and Generation of the 3-D Deformation Field

Intensity-based registration algorithms are prone to be affected by the poor quality of CBCT images. In general, there is a large intensity gradient at the boundary of the tissue or organ. This is especially true for images of the chest. Gradient-based registration can avoid the registration error induced by the inaccuracy of CBCT intensity. The GFFD algorithm is based on the theory that although the portion of the total signal intensity caused by scattered radiation can account for up to 50% or more (without anti-scatter grids), it is generally homogeneous. The shapes of most inner and outer object boundaries can be perceived in the scatter artifact images (15). In the previous study, we compared the Demons registration with GFFD registration using the evaluation framework presented by Martin Urschle (16). Compared to the Demons registration, GFFD registration has a 43.4% reduction in the mean absolute difference and a 66.7% reduction in the mean absolute error of the edge. Based on these results, we can conclude that the GFFD algorithm is more accurate and robust compared to the Demons algorithm in deformable registration of CBCT and CT images. Details of the GFFD algorithm are described elsewhere (14).

The corrected CT is generated by registering the planning CT and the CBCT of the day using the GFFD algorithm. Due to the inaccuracy of electron density in CBCT images, CBCT cannot be directly used for dose calculation. We mapped the electronic density information of the planning CT to the CBCT through the deformation field generated from the registration. The corrected images not only reflect the current tumor and OARs positioning, but also have accurate electronic density. Image registration includes two steps: the first step is to perform the rigid alignment between the planning CT and the CBCT; the second step is to generate voxel-to-voxel correspondence between the planning CT and CBCT image and to obtain the 3-D deformation field between the two.

In order to reduce the manual delineation time, propagation of the target area and organs to the corrected CT image was performed automatically according to the deformation field generated by registration. The automatic delineation was divided into the following steps: (1) filling the contours of the organs on the planning CT slice by slice using the flood filling algorithm; (2) obtaining corresponding deformation of each organ from CBCT to planning CT according to the deformation field from planning CT to CBCT; and (3) re-extracting the outline of the filled organ on the CBCT by the matching cube algorithm. The process of image registration and automatic delineation was completed by the GFFD algorithm. In this registration algorithm, we used a multi-resolution registration strategy to make the registration process faster and to avoid the local minimum value. The validation for the new segmentation was given in Appendix A (Supplementary Material).

### Acquisition of the 2-D Deformation Field in BEV

**Figure 2:**
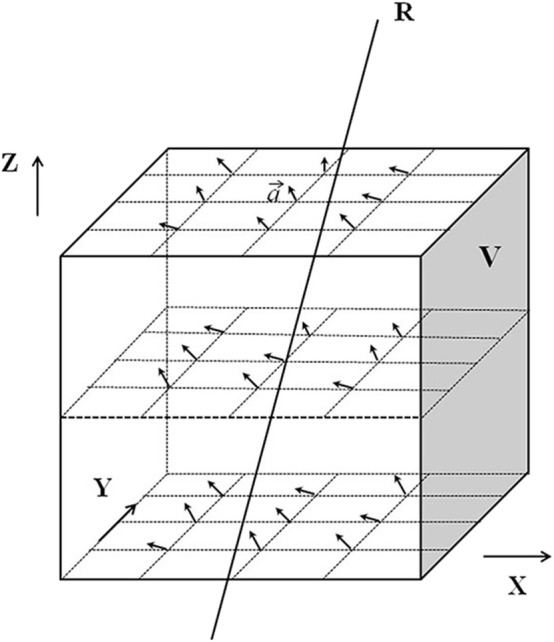
Schematic diagram of obtaining the two-dimensional deformation vector. V represents the ROI set by the X, Y, and Z axes of the coordinate system. The arrows in the ROI V represent the 3D deformation vector “$\vec{a}$” of each pixel in the ROI.

The 2-D deformation vector field of radiation intensity in each beam's eye view (BEV) in the original plan was determined according to the 3-D deformation vector field generated by image registration in the ROI. ROI refers to the region of the target or surrounding OARs. The determination of the 2-D deformation vector field from the 3-D deformation vector field is illustrated in Figure 2. The coordinate system direction is consistent with that of the gantry coordinate system in the International Electronic Commissioning IEC-61217 coordinates. It is stationary with respect to the beam limiting device, whose origin is the isocenter. In this coordinate system, the axis Z was coincides with direction of beam. The axis X and Y are perpendicular to the radiation field.

The deformation vector “$\vec{a}$” can be expressed as:

(1) $$\begin{array}{l} \vec{\text{a}}\;=\left[\begin{array}{ccc} {\text{x}}_{\text{t}} & - & {\text{x}}_{\text{o}}\\ {\text{y}}_{\text{t}} & - & {\text{y}}_{\text{o}}\\ {\text{z}}_{\text{t}} & - & {\text{z}}_{\text{o}} \end{array}\right] \end{array}$$

Where (**x**_**t**_**, y**_**t**_**, z**_**t**_) is the end point of $\vec{a},\;({\text{x}}_{\text{o}},{\text{y}}_{\text{o}},{\text{z}}_{\text{o}}$*)* is the starting point of $\vec{a}$, so $\vec{a}$ is represented by the difference between the starting point and the end point. In Figure 2, R is a ray passing through the ROI V. The deformation vectors of the pixels crossed by R are $\vec{a1}$,$\vec{\;a2}$*, ……*$\vec{an}$, $\vec{a{\;}^{\prime }}$is the projection of $\vec{a}$ on the isocenter plane, the projection of $\vec{a1}$,$\vec{\;a2}$, ……$\vec{an}$ onto the isocentric plane are denoted as:

(2) $$\begin{array}{l} \vec{\text{a}1^{\prime }}\text{=}\left[\begin{array}{ccc} {{\text{x}}_{\text{t}1}}^{\prime } & - & {{\text{x}}_{\text{o}1}}^{\prime }\\ {{\text{y}}_{\text{t}1}}^{\prime } & - & {{\text{y}}_{\text{o}1}}^{\prime }\\ {{\text{z}}_{\text{t}1}}^{\prime } & - & {{\text{z}}_{\text{o}1}}^{\prime }\\ \; \end{array}\right]\\ \vec{\text{a}2^{\prime }}\text{=}\left[\begin{array}{ccc} {{\text{x}}_{\text{t}2}}^{\prime } & - & {{\text{x}}_{\text{o}2}}^{\prime }\\ {{\text{y}}_{\text{t}2}}^{\prime } & - & {{\text{y}}_{\text{o}2}}^{\prime }\\ {{\text{z}}_{\text{t}2}}^{\prime } & - & {{\text{z}}_{\text{o}2}}^{\prime }\\ \; \end{array}\right]\\ \text{ ……}\\ \vec{{\text{an}}^{\prime }}\text{=}\left[\begin{array}{ccc} {{\text{x}}_{\text{t}\text{n}}}^{\prime } & - & {{\text{x}}_{\text{o}\text{n}}}^{\prime }\\ {{\text{y}}_{\text{t}\text{n}}}^{\prime } & - & {{\text{y}}_{\text{o}\text{n}}}^{\prime }\\ {{\text{z}}_{\text{tn}}}^{\prime } & - & {{\text{z}}_{\text{on}}}^{\prime } \end{array}\right] \end{array}$$

*x*′, *y*′, *z*′are denoted as:

(3) $$\begin{array}{l} {\text{x}}^{\prime }\text{=}\frac{\text{x SAD}}{\text{SAD - z}} \end{array}$$

(4) $$\begin{array}{l} {\text{y}}^{\prime }\text{=}\frac{\text{y SAD}}{\text{SAD - z}} \end{array}$$

(5) $$\begin{array}{l} {\text{z}}^{\prime }=0 \end{array}$$

where SAD is the source-axis distance.

### Adjustment of the 2D Radiation Intensity Distribution

According to the 2-D deformation vector field, 2-D radiation intensity distribution in the corresponding beam direction in the original plan was deformed to generate a new 2-D radiation intensity distribution in each beam direction.

**Figure 3:**
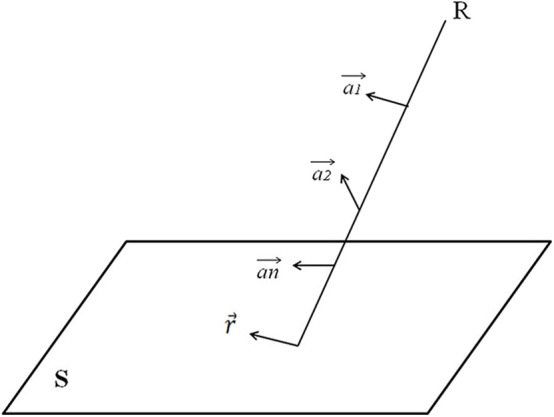
Explanation of how to obtain the ray projection $\vec{r}$ on the isocentric plane. S is an isocenter plane, and S is parallel to the plane in which the coordinate system X and Y axis are located. Each point on S (i.e., the intersection of the ray R and the isocenter plane S) represents a ray. The intensity of each ray is I, and there is a 2-D ray intensity distribution I (x, y) on the isocenter plane.

Figure 3 demonstrates how to obtain the ray projection $\vec{r}$ on the isocentric plane. The equation for projection $\vec{r}$, corresponding to R on the isocentric plane S, is:

(6) $$\begin{array}{l} \vec{\text{r}}\text{=}(\vec{\text{a}1^{\prime }}+\vec{\text{a}2^{\prime }}+\ldots +\vec{{\text{an}}^{\prime }})/\text{n} \end{array}$$

Using the above equation, the 3-D deformation vector field can be defined as the 2-D deformation vector field of ray intensity on the isocentric plane. Then, the 2-D ray intensity distribution I (x,y) on the isocentric plane is deformed along the 2-D deformation vector field r to generate a new 2-D ray intensity distribution I ′(x′,y′). According to the new 2-D ray intensity distribution in each beam direction, the corresponding MLC and jaw motion data are generated, and the new radiotherapy plan is obtained.

### Testing Cases

#### Plan Comparisons

In this proof-of-principle study, the effectiveness of the method was demonstrated and tested in 20 consecutive patients receiving curatively intended RT for lung tumors in the thoracic region. The clinicopathologic characteristics of the patients were presented in Table 1. Stage was defined according to the American Joint Committee on Cancer (AJCC, 7th edition) staging system.

**Table 1:** Characteristics of the patients.

**Characteristic**	***n***
**Gender**	
Male	16
Female	4
**Lateral**	
Left	13
Right	7
**The American Joint Committee on Cancer stage (7**^**th**^ **edition)**	
IB	3
IIA	2
IIB	3
IIIA	8
IIIB	4

The target area of central lung cancer patients included metastatic lymph nodes. The original plans for the lung cases were designed to deliver a dose of 60 Gy in 30 fractions for each case. The clinical target volume (CTV) was defined as the gross tumor volume with a 5 mm radial margin extension and the involved lymph node drainage area. The planning target volume (PTV) was defined as the CTV adding a 5 mm margin in all directions. The original plans were designed based on appropriate dose–volume constraints for both target and OARs. The CBCT images were acquired weekly during radiation treatment for the patients. All CBCT images were acquired during the routine IGRT using an imaging system equipped on the Varian Trilogy linear accelerator machine.

#### Plan Evaluations

To evaluate the feasibility of our method, three different sets of dose-volume data were compared for each case: (1) the original plan: the original IMRT plan applied on the corrected CT images with the isocenter determined from skin marks. This represented the conventional treatment; (2) the repositioning plan: the original IMRT plan applied on the corrected CT images with isocenter determined to achieve the maximum overlap between the new and the old CTVs. This represented the current common practice of IGRT; and (3) the IFP plan: the original IMRT segments modified by our method based on the corrected CT image.

Paired two-tailed Wilcoxon signed-rank tests (SPSS, version 17.0 software; SPSS Inc., Chicago, IL) were performed to compare the dosimetric parameters for the three scenarios. Any results with a *P* ≤ 0.01 were considered statistically significant.

## Results

With GPU acceleration, the time frame of image registration and automatic organ mapping was about 30 s. The entire replanning process took about 3 min for using one GPU card in all tested lung cancer cases. For clarity, a detailed comparison between the state of art algorithms and our method is summarized in Table 2.

**Table 2:** Comparison between the IFP and other algorithms.

**Method**	**Concept**	**Automated/ semi-automated**	**Time frame**	**Site of the sample patient cases**	**Key parameters**	**Limitations**
SAM+SWO (6)	Morphing beam segment shapes to match the new location and shape of the target; Optimizing the new segment weight	Semi-automated	Within 10 min	Two prostate canses; one pancreas case	V100% = 98% for GTV	Requires the contours of important structures to be drawn online
Method by Li et al. (17)	Iteratively adjusting voxel-weighting factors in an objective function under the guidance of DVHs	Automated	30 s	Three head-and- neck cases	D99% = 58–69 Gy, V95% = 101–102% for PTV70; max cord dose = 13–24 Gy, max brainstem dose = 13–19 Gy, left mean parotid dose = 8–14 Gy, right mean parotid dose = 15–20 Gy	The achievability of the original DVHs in the new patient geometry will impact the efficacy of the algorithm; neglects the spatial dose information
GM (18)	Capture the dose gradients from the original plan; Proceeds with a replanning optimization process aiming to maintain the originally achieved dose gradients on the anatomy of the day based on the daily image	Automated	Within 5 min	Five prostate and Five pancreas casess	Prostate: D95% = 75.6 Gy, mean bladder dose = 23.0 ± 9.9 Gy, max femoral-head dose(left) = 39.8 ± 7.7 Gy, max femoral-head dose(right) = 38.1 ± 9.9 Gy; Pancreas: D95% = 50.4 Gy, mean duodenum dose = 21.9 ± 4.7 Gy, mean stomach dose = 4.0 ± 2.9 Gy, mean liver dose = 3.1 ± 2.0 Gy, max cord dose = 20.7 ± 7.4 Gy	Not suitable for the case of large OAR deformation
Method by Zarepisheh et al. (19)	Creates a treatment plan guided by the DVH curves of a reference plan that contains information on the clinician-approved dose-volume trade-offs among different targets/organs and among different portions of a DVH curve for an organ	Automated	~ 10 s	Two prostate case; one head-and-neck case	Prostate: D95% = 71 Gy, max femoral-head dose = 30 Gy, max bladder dose = 75 Gy, max rectum dose = 78 Gy; Head-and-neck: D95% = 70 Gy for PTV70, max cord dose = 36 Gy, max brainstem dose = 30 Gy	Only pick the DVH to guide the process while other clinical related factors were ignored
IFP	Realizing adaptive correction by projecting a 3-D vector field into a 2-D vector field for deformation of intensity distribution	Automated	Approximately 3 min	Twenty lung cases	V100% = 100%, D95% = 60 Gy for CTV; mean total lung doses = 12.2 Gy, mean heart doses = 4.7 Gy, max cord dose = 38.5 Gy	Not sensitive to the site with low gray-gradient

Table 3 shows the metrics used to compare the quality among the original, IFP and repositioning plans for the 20 lung cancer cases. Average dosimetric parameters for the three plans are presented. Substantial underdosing in the CTV is seen for the original and the repositioning plans in several fractions. *D*95 average values were 44.0 and 58.5 Gy, respectively, which were less than the prescribed dose of 60 Gy. The IFP plan met the prescription dose requirements (on average, CTV D95 for IFP plan is 60.3 Gy) which were superior to the original and the repositioning plans in CTV coverage. The difference was statistically significant. The value of V100% showed the same result. For most plans, CTV coverage was highest on the optimization plan followed by the repositioning plan, while the CTV coverage of the original and repositioning plans was unacceptable. According to Table 3, there was no difference between the HI values of the original and the repositioning plans, while the HI values of the IFP plan were significantly lower than those of the other two plans, indicating that the dose uniformity of the IFP plan was better than the other two plans.

**Table 3:** Average dosimetric parameters for the three scenarios (original plan, repositioning plan, and IFP plan) of the lung cases.

	**Average**	**Original:1**	**Repositioning:2**	**IFP:3**	**p:1-2**	**p:1-3**	**p:2-3**
CTV	D95 (Gy)	44.0	58.5	60.3	0.49	<0.01	<0.01
CTV	V100%	82.6	86.7	100	0.17	<0.01	<0.01
CTV	HI (D5/D95)	1.3	1.2	1.1	0.20	<0.01	<0.01
Cord	Max (Gy)	38.6	38.5	38.5	0.76	0.95	0.93
Total lung	Mean (Gy)	12.4	12.6	12.2	<0.01	<0.01	<0.01
Total lung	V5 (Gy)	45.1	44.8	44.6	<0.01	<0.01	0.01
Total lung	V20 (Gy)	20.0	19.7	20.2	0.94	0.4	0.15
L-lung	Mean (Gy)	9.2	9.3	9.0	0.81	<0.01	<0.01
L-lung	V5 (Gy)	40.1	39.4	39.3	0.01	<0.01	<0.01
L-lung	V20 (Gy)	13.4	12.9	13.7	0.02	0.79	0.55
R-lung	Mean (Gy)	16.6	16.5	16.0	0.67	0.09	0.06
R-lung	V5 (Gy)	51.8	52.6	53.4	0.35	0.14	0.12
R-lung	V20 (Gy)	29.1	29.2	29.4	0.27	0.65	0.87
Heart	Mean (Gy)	5.6	5.4	4.7	0.05	<0.01	<0.01
Heart	V30 (Gy)	14.2	15.0	12.7	<0.01	<0.01	<0.01

*CTV, clinical target volume; D95, the doses with which 95% of the CTV was covered; V100%, the percentage of the CTV receiving the prescribed dose; HI, homogeneity index; V5, the percentages of the normalized volume of OARs receiving 5 Gy; V20, the percentages of the normalized volume of OARs receiving 20 Gy; V30, the percentages of the normalized volume of OARs receiving 30 Gy*.

In terms of the dose parameters of OARs, all three scenarios met the dose limitation requirements of the Quantitative Analysis of Normal Tissue Effects in the Clinic (QUANTEC) standards. In general, the OARs doses of the IFP plans were lower than or equivalent to the original and the repositioning plans, as shown in Table 3. The repositioning plan also resulted in lower OARs doses compared to the original plan, but the difference was smaller than the difference between the IFP and the original plan. On average, the mean total lung doses were 12.2 Gy for the IFP plan compared to the 12.4 and 12.6 Gy for the original and repositioning plans. However, the doses of the IFP plans were not different from other plans on the V20 of total lung. Similar trend was found in the left lung, where the average mean dose in the IFP plan was significantly (*P* < 0.01) lower than the original and repositioning plans. However, the V20 to the left lung among the three sets of plans showed no statistical difference. For mean dose of the heart, the IFP plans were lower than the other plans as well, while there was no difference between the original plans and the repositioning plans, which indicated that the IFP plan could improve heart sparing. The IFP plan did not show any improvement for sparing of the right lung, but this plan satisfied our constraint (maximum dose <45 Gy).

**Figure 4:**
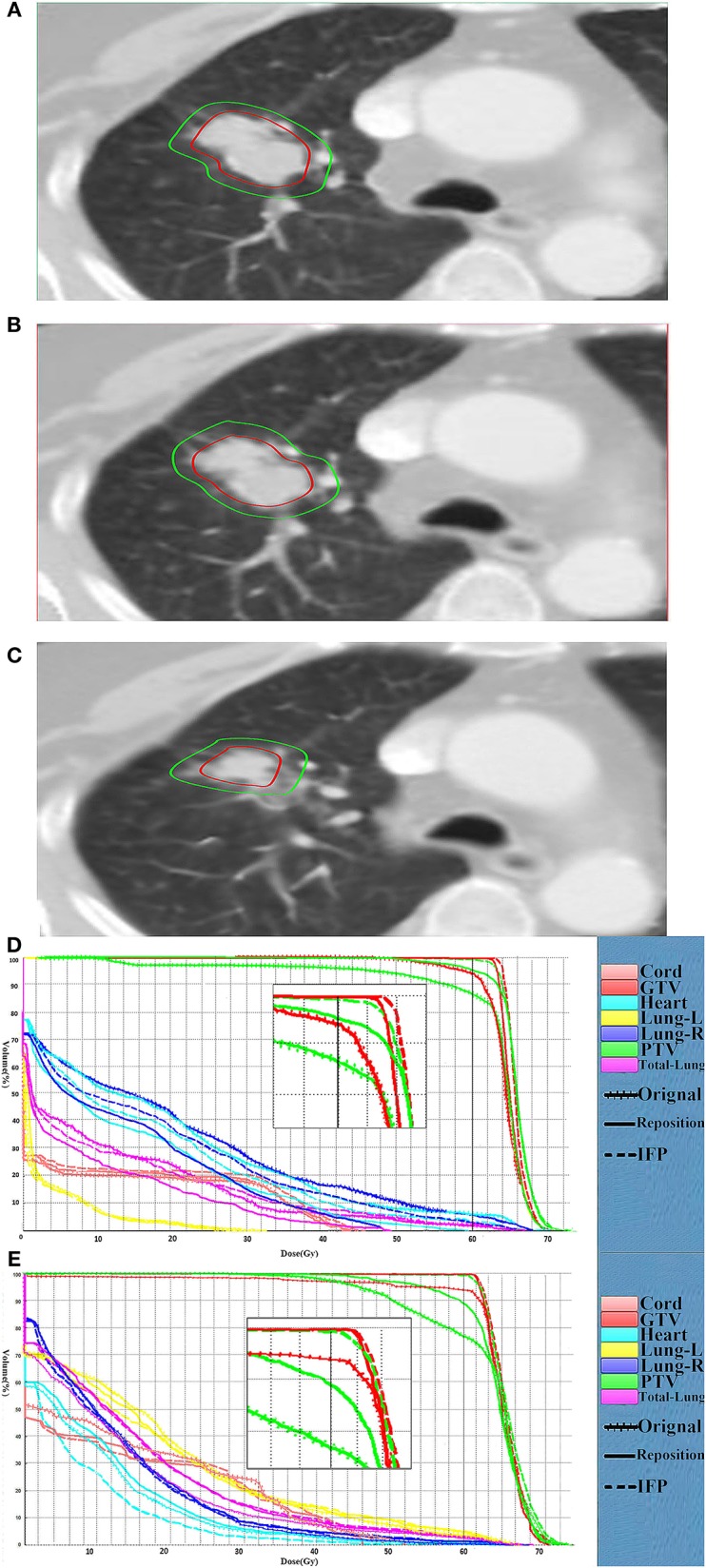
Comparison of the target volume in the representative CT slice. Comparison of the target volume in the representative CT slice obtained on the CT-plan **(A)**, the corrected CT of fraction 6 **(B)** and the corrected CT **(C)** of fraction 16, and DVH curves comparison for the fraction 6 **(D)** and fraction 16 **(E)** for patient 1. The dotted, dashes, and solid curves represent the original, IFP and the repositioning plans, respectively.

The comparison of target volume and DVHs on representative CT slices of a lung cancer case (patient 1) was shown in Figure 4. Compared with the planning CT, the deformation of target volume in the image of fraction 6 is minimal, while the deformation in fraction 16 is significant. Figures 4D,E present the DVHs obtained under the three scenarios of 6th and 16th fractions. As can be seen, the target volume would be significantly underdosed if using the original plan. As the treatment continued, this underdosing became more pronounced due to the increased deformation in the target area. Compared with the original plan, the dose distribution can be improved by repositioning, but this is not enough to meet the treatment goal. These results confirmed that our algorithm is able to automatically generate an adaptive plan that has improved target coverage and OARs sparing compared to the repositioning plan for the patient's new geometry.

**Figure 5:**
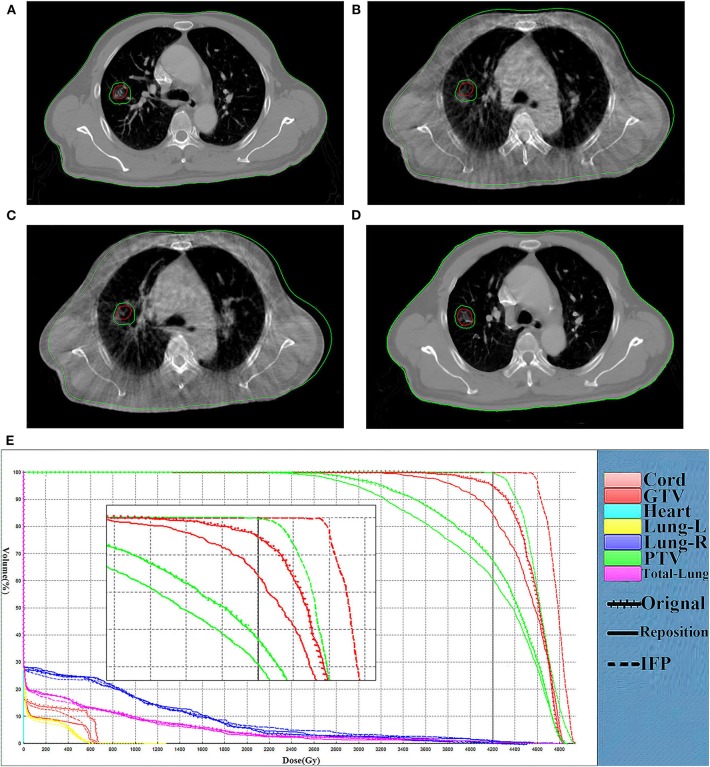
Comparison of representative CT slices. Comparison of representative CT slices obtained on the planning CT **(A)**, the CBCT before shift couch **(B)**, the CBCT after shift couch **(C)**, and the corrected CT image **(D)** as well as DVH curves comparison **(E)** of the same fraction for a lung cancer patient. The dotted, dashes, and solid curves represent the original, IFP and the repositioning plans, respectively.

In the research process, some patients were observed to have an increase in setup error after rigid body registration. Furthermore, the dose distribution generated by repositioning according to this rigid body registration was seriously inaccurate. Figure 5 shows the comparison of representative CT slice obtained on the planning CT (Figure 5A), the CBCT before shift couch (Figure 5B), the CBCT after shift couch (Figure 5C), and the corrected CT image (Figure 5D) for a lung cancer patient. Figure 5 demonstrates that the lesion would be significantly underdosed if it was treated without repositioning (Figure 5B), and this underdosing was more pronounced after rigid body registration (Figure 5C). This geographic missing was corrected after deformable registration (Figure 5D). This geographic missing resulted in target volume underdosing as indicated by the DVH in Figure 5E. The repositioning plan was worse than the original plan in terms of CTV coverage. The IFP algorithm was sufficient to yield good CTV coverage without increasing the exposure dose of OARs.

## Discussion

We have successfully developed an online adaptive replanning algorithm to rapidly generate a new plan adapted to the patient changed geometry. The automated organs propagation and segment adjustment, avoid tedious trial-and-error procedure, and enable online replanning. To verify the effectiveness of our method, we tested the method on 20 patients and observed promising results. The IFP plans are much better in terms of CTV coverage and OARs sparing compared with the conventional and repositioning plans with significantly reduced manual labor. The overall replanning process takes <3 min.

A key issue regarding the online replanning strategy is whether it can be implemented with realistically acceptable accuracy and time frame. A variety of online replanning methods have been developed previously to generate adaptive plans in a timely manner. However, planning optimization is a complicated process. Different planning systems have different objective functions, and optimization results are affected by many parameters. Even if the parameters are unchanged, small changes in the anatomy can lead to completely different optimization results. Li et al. (17) developed an automatic replanning algorithm to generate plans with DVH curves that are similar and possibly better than the original plans. They iteratively adjust the voxel-weight factor to achieve an automatic replanning process, but this is an open-loop process which requires a stopping strategy to end the iteration. Ahunbay and Li (18) proposed an algorithm which can reproduce the dose gradients based on the original plan during the process of replanning. This method assumes that any point on the surface of the daily CT target volume surface has a corresponding point on the surface of the CT target volume. However, when the target volume has numerous variations, the two target surfaces may not be able to establish a point-to-point relationship. Mohan et al. (20) also suggested an online correction method that deformed the intensity distributions for each beam based on the deformation of structures seen in the BEV. This approach is based on the assumption that the overlapping parts of each ROI on each CT have a one-to-one correspondence. However, the overlapping area of each ROI may disappear during actual radiotherapy, so it is not necessarily a one-to-one correspondence. The IFP algorithm would be less affected compared to other online re-optimizing methods since it directly adjusts the intensity distribution based on the deformation field.

The implementation of the online replanning algorithm relies on rapid segmentation. The use of DIR to automate target and OARs contouring is one solution to reduce the time and human labor required for online ART. DIR shows promise in automating the contouring process by propagating planning contours to daily images and reducing the amount of time to delineate organs (1, 2). In this case, physicians must review and possibly edit any contours generated from DIR algorithms. Even so, the DIR algorithm should reduce the processing time. A number of deformation registration algorithms have been developed to register images and generate target regions, such as the Demons algorithm (21), the contour-guided deformable image registration algorithm (22) and the B-spline-based registration (23). These algorithms can perform the deformation registration of CBCT images and CT images. However, the inherent electron scattering of CBCT will affect the quality of the reconstructed images and the electron density, which will directly reduce the registration accuracy (24, 25). The presently proposed IFP method avoids the inaccurate matching of CBCT image density by correcting CBCT pixels.

Other obstacles to executing optimization include setting multiple dose limits and continuously performing trial-and-error tests to reach a balance between the target dose and the OARs dose. This process requires a certain amount of time and does not meet the requirements of online replanning. The IFP method directly changes the original field setting through the deformation field, thus avoiding the problems caused by the trial-and-error test.

The algorithm proposed in this paper is based on the assumption that there is a significant density gradient between different ROI boundaries. The algorithm used may reduce this concern and work for a tumor site with high image contrast. However, boundaries with small gradient also exist in practical situations, which would lead to inaccuracy in DIR and subsequently the deformed vector field. The results of the DIR of this study were desired, probably because of the use of lung images. Tumor site with smaller border gradients such as prostate or head and neck will be used to further test the proposed algorithm.

For fractions with little or no deformation, the DIR will not be required. As shown in the results, the proposed scheme is more suitable for fractions when significant deformation is present in the middle and late stages of radiotherapy. More rigorous studies are needed to screen patients who could benefit from the replanning method.

## Conclusion

A fast online replanning algorithm to correct for interfractional anatomy variations was presented. This algorithm adjusts the segment according to the deformation field generated by DIR, and only requires a small amount of manual delineation. Therefore, this can be implemented within a practically acceptable time frame. Our preliminary tests proved that the adaptive plan obtained from this replanning method is superior to the original plan and the repositioning plan.

## Data Availability Statement

The datasets generated for this study are available on request to the corresponding author.

## Ethics Statement

The studies involving human participants were reviewed and approved by Shandong Cancer Hospital and Institute. The patients/participants provided their written informed consent to participate in this study.

## Author Contributions

XLiu analyzed the experimental raw data, and was a major contributor in writing the manuscript. YL and BL designed the experiment. GY executed the experiment process and recorded the data. XLi revised the manuscript for important intellectual contents. QC and YY checked the experimental raw data. JZ helped to collect the clinic data. All authors read and approved the final manuscript.

### Conflict of Interest

YL was employed by the STFK Medical Device Co. The remaining authors declare that the research was conducted in the absence of any commercial or financial relationships that could be construed as a potential conflict of interest.
