# Supplementary material for: A Fast Online Replanning Algorithm Based on Intensity Field Projection for Adaptive Radiotherapy
Source: Front Oncol. 2020 Mar 3;10:287. doi: 10.3389/fonc.2020.00287 (PMC7063069; doi:10.3389/fonc.2020.00287)
Supplement: Supplementary file 1 [file Data_Sheet_1.docx]

**Appendix A. Accuracy validation for the new segmentation**

Due to the limitations of DIR, human intervention is necessary at least for validation of the contours. We randomly selected 15 lung cancer patients. Weekly CBCT scans were acquired for each patient during the course of radiotherapy. Generate the corrected CT by registering the planning CT and the CBCT with the GFFD algorithm. Three delineation modalities were applied on the same set of CT images: auto-contours by the GFFD algorithm, contours propagated based on rigid registration using the center of volume of the lesion and manual contours by four radiation oncologists with expertise in lung cancer. We calculated the Dice similarity coefficients (DSCs), Hausdorff Distance (HD) and the root mean square (RMS) surface distance between the manual-segmentation and auto-segmentation and compared those with the same metrics between the manual-segmentation and segmentation propagated based on rigid registration. The value of DSC represents the volume overlap between two sets of segmentations, the HD and RMS distances represent the maximum and average surface distances of the two segmentations, respectively. DSC value is equal or greater than 95% represents a high volume overlap of segmentations. When the value of DSC is lower than 95%, the automatically delineated target area needs manual modification, according to the modified target area, the deformed vector field is also modified through interpolation algorithm.

  Paired-sample *t*-tests (SPSS, version 17.0 software; SPSS Inc, Chicago, IL) were performed to compare the metrics between auto-contour and contours propagated based on rigid registration. Any results with a *P* value ≤0.01 were considered statistically significant.

**Table S 1** Dice similarity coefficient (DSC), Hausdorff distance (HD) and root mean square (RMS) statistics for different segmentations on the gross tumor volume in the 15 test cases

| Patient | DSCs (%) | |  | HD(mm) | | |  | | RMS(mm) | | |
| --- | --- | --- | --- | --- | --- | --- | --- | --- | --- | --- | --- |
|  | Auto-contours | Rigid |  | Auto-contours | | Rigid |  |  | Auto-contours | | Rigid |
| 1 | 96.04±0.52 | 96.78±1.23 | | | 3.83±0.81 | 3.52±1.81 | | 3.19±0.12 | | 4.76±0.10 | |
| 2 | 95.83±0.51 | 91.01±1.61 | | | 4.80±0.63 | 6.73±1.91 | | 3.72±0.26 | | 4.62±0.14 | |
| 3 | 90.94±1.72 | 87.28±0.75 | | | 6.09±1.25 | 8.93±1.43 | | 3.74±0.19 | | 3.54±0.12 | |
| 4 | 95.89±0.78 | 92.89±1.04 | | | 4.32±1.12 | 5.84±1.80 | | 3.44±0.14 | | 3.21±0.16 | |
| 5 | 91.10±0.86 | 90.25±0.99 | | | 5.86±1.42 | 6.08±1.86 | | 4.72±0.22 | | 4.79±0.23 | |
| 6 | 89.92±0.69 | 87.30±0.5 | | | 9.18±0.80 | 9.69±2.20 | | 4.21±0.11 | | 4.89±0.11 | |
| 7 | 90.08±1.01 | 85.28±0.69 | | | 8.35±1.13 | 10.21±1.41 | | 4.91±0.17 | | 6.11±0.11 | |
| 8 | 96.04±1.03 | 94.86±2.33 | | | 4.12±0.46 | 5.16±3.62 | | 3.45±0.17 | | 4.35±0.28 | |
| 9 | 96.67±0.69 | 91.85±1.16 | | | 4.13±0.66 | 6.76±1.89 | | 3.02±0.24 | | 3.78±0.12 | |
| 10 | 94.45±0.43 | 92.43±0.82 | | | 5.99±0.46 | 7.41±3.01 | | 3.42±0.43 | | 3.08±0.15 | |
| 11 | 96.70±0.77 | 95.66±1.20 | | | 3.01±1.17 | 5.25±0.28 | | 2.22±0.05 | | 2.15±0.17 | |
| 12 | 96.62±0.76 | 94.33±0.66 | | | 4.78±0.61 | 6.71±1.52 | | 3.70±0.13 | | 3.48±0.22 | |
| 13 | 96.58±0.83 | 93.45±0.53 | | | 4.97±0.90 | 7.68±1.91 | | 3.61±0.10 | | 3.58±0.14 | |
| 14 | 95.58±1.06 | 94.01±2.03 | | | 5.31±1.22 | 6.30±1.54 | | 4.05±0.24 | | 3.98±0.13 | |
| 15 | 95.71±1.55 | 95.55±1.29 | | | 5.34±1.26 | 5.82±2.23 | | 3.89±0.18 | | 3.96±0.15 | |
| P | ＜0.01 | | | | ＜0.01 | | | 0.053 | | | |

The table compares means ±SD of Dice’s similarity coefficient values (DSC), the root mean square (RMS) surface distance and the Hausdorff Distance (HD) for GTV between manual-segmentations and auto-segmentations of the lesion and those between the manual-segmentations and for contours propagated based on rigid registration using center of the volume fifteen patients.

Figure S1 shows an example of representative slices of different physician manual segmentation of GTV and auto-segmentation on the same slice. The similarity of the target volume enclosed by the three segmentation modalities can be seen from the DSC values, HD and RMS distance values shown Table S1. The DSCs between auto-segmentation and manual-segmentation was ranging from 84.41% to 98.95 %, significantly higher than the agreement between contours propagated based on rigid registration and manual-contours. The two distance-based metrics showed the same trend to DSC values, the mean HD values of the GTV were ranging from 2.36mm to 11.26mm. The average value of HD is also lower than the contours based on rigid registration indicating that auto-segmentation were more acceptable in this study.


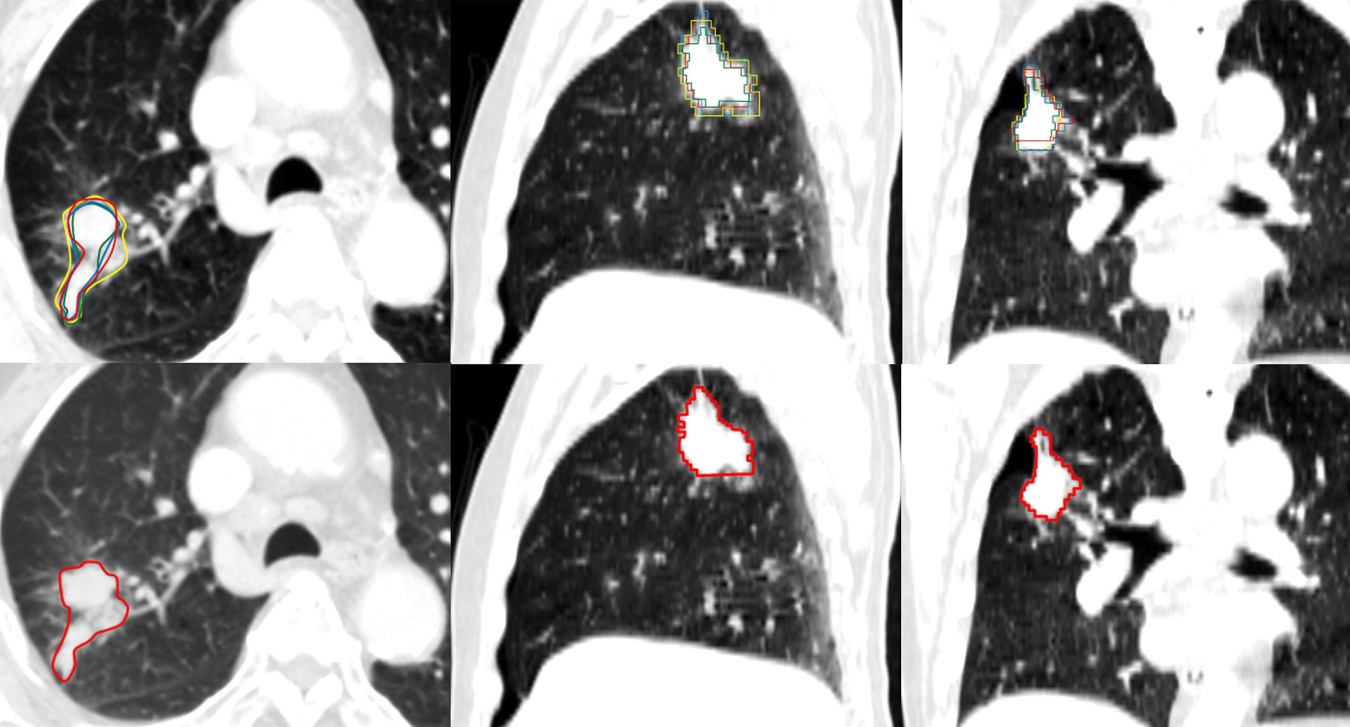


Figure S1. Example of representative slices of 4 physicians’ manual segmentation GTV and auto-segmentation on the same slice in the axial, sagittal, and coronal image planes. Top panels represent multiple physicians’ manual segmentations with each color representing a different observer. Bottom panels represent the auto-segmentations.
